# Microbiome and Asthma: What Have Experimental Models Already Taught Us?

**DOI:** 10.1155/2015/614758

**Published:** 2015-07-22

**Authors:** R. Bonamichi-Santos, M. V. Aun, R. C. Agondi, J. Kalil, P. Giavina-Bianchi

**Affiliations:** Clinical Immunology and Allergy Division, University of São Paulo School of Medicine, 01454-010 São Paulo, SP, Brazil

## Abstract

Asthma is a chronic inflammatory disease that imposes a substantial burden on patients, their families, and the community. Although many aspects of the pathogenesis of classical allergic asthma are well known by the scientific community, other points are not yet understood. Experimental asthma models, particularly murine models, have been used for over 100 years in order to better understand the immunopathology of asthma. It has been shown that human microbiome is an important component in the development of the immune system. Furthermore, the occurrence of many inflammatory diseases is influenced by the presence of microbes. Again, experimental models of asthma have helped researchers to understand the relationship between the microbiome and respiratory inflammation. In this review, we discuss the evolution of murine models of asthma and approach the major studies involving the microbiome and asthma.

## 1. Introduction

Asthma is a common and potentially serious chronic inflammatory disease that imposes a substantial burden on patients, their families, and the community [1]. It is recognized as a complex disease with a heterogeneous profile divided into phenotypes. The phenotypes can be distinguished by their pathology, immunology, clinical manifestations, response to treatments, and long-term outcomes [2]. Asthma is one of the most common chronic diseases worldwide and its global economic cost exceeds those of tuberculosis and HIV/AIDS combined [3, 4]. Globally, about 300 million people are currently receiving treatment for the disease.

Unfortunately, 5 to 10% of patients present severe asthma, which remains uncontrolled despite high doses of inhaled corticosteroid combined with long-acting beta-agonists [5]. Even corresponding with a small proportion of patients, severe asthma represents the majority of asthma-related deaths and almost half of the total healthcare costs associated with the disease [6, 7].

Less than 12% of drugs developed for use in humans are successfully registered and there are still high costs for developing these drugs to enter the market [8, 9]. Experimental models are important for the study of asthma immunopathogenesis and as a template for new therapies development.

## 2. Experimental Models in Asthma

There are three types of experimental models in asthma that are frequently used in research:* in silico*,* in vitro*, and* in vivo*.* In silico* models are performed on computers and can simulate diseases and generate virtual patients. Human cells can be studied* in vitro* in physiological and pathological conditions. However, this model is not suitable to assess environment influences and long-term effects. The third and most commonly used type of experimental model in asthma is* in vivo* model, particularly the murine model.

## 3. *In Vivo* Asthma Models

Animal models have been used for over 100 years in mechanistic studies, increasing the scientific knowledge of asthma [10]. Researchers have studied abnormalities which are characteristic of asthma, such as airway flow limitation, inflammation, and remodeling. Animal models allow us to test the efficacy of a particular drug as well as its toxicity before starting clinical trials for safety and efficacy in humans. However, the interpretation and extrapolation of the results originating from such models for patients with asthma are highly dependent on the outcome of interest and animal species chosen in the experimental model [11].

No experimental animal is known to spontaneously develop a disease with characteristics that can be considered to be asthma [12]. However, there are some examples of asthma-like disease in the animal kingdom: cats can present a bronchial disease that is similar to human chronic asthma [13] and horses can develop a neutrophil dominated airway disease known as “heaves” that has some of the hallmarks of asthma [14].

The development of animal models of allergic asthma involves sensitization of the animal to an antigen of interest and subsequently challenge of the airways, triggering an allergic response [14]. Many species are used as models of asthma, such as guinea pig, rat, and mouse, which are the most commonly used. Unfortunately, none of them can be considered an ideal murine model [15].

Guinea pigs are able to cough and sneeze, and pulmonary changes presented by them after exposure to allergens are similar to changes in FEV1 after bronchial challenge in humans. Furthermore, the lung anatomy of guinea pigs is more similar to human lung compared to mice. On the other hand, transgenic or knockout animals are not available and reagents have restricted availability. In addition, sensitization of airways and response to allergen may vary over time and between laboratories and depending on the source of animals [15, 16].

Rats have some advantage over mice for the study of inhaled agents, but again reagents and transgenic or knockout animals are not readily available. The airway response to allergens may vary over time and there are also variations between different laboratories and animal sources [15, 16].

Mice are relatively easy to create and procreation is fast. In addition, chemical reagents are widely available, as well as knockout animals and transgenic strains. Unfortunately, it is difficult to extrapolate doses of inhaled allergen from mouse models to humans. In addition, lung anatomy of these animals is very different from human lung (e.g., small size, less bifurcations), as well as lung biology/pharmacology (e.g., absence of *β*2-adrenergic receptors in the smooth muscle of mice airway) [15, 16].

## 4. Research on Mice Models of Asthma

Mice have become the most commonly used species because they are easier to create, procreate, maintain, and handle. There is a wide variety of reagents available for determination of antibodies and cytokines, as well as transgenic or knockout strains, which are used to study the mechanisms of immune diseases [15].

There are well-documented differences in lung anatomy/histology of humans and mice, such as the organ location, branching airways, blood supply, smooth muscle, and cell types. These factors can affect the inflammatory response and lung function that are regularly evaluated in asthma studies [16–18].

Many questions about human asthma are still unanswered. There is a broad understanding of the Th2 response in atopic asthma, but researchers still do not understand what happens to about 50% of patients with nonallergic asthma [19]. Furthermore, there are few therapeutic options available for the treatment of patients who do not respond to inhaled corticosteroids (ICS) [20]. It is urgent to understand the Th2-low subsets, noneosinophilic asthma [21] and develop therapies for these patients, especially for those with severe asthma [22, 23]. Good murine models of nonallergic asthma are still missing. In this context, more translational studies must be done and new experimental models must be developed [24].

Asthma mouse models have changed significantly since the 1990s. Latest models assessed the role of relevant risk factors and evaluated potential drug targets. Furthermore, studies have demonstrated the role of various cells and tissues in lung inflammation, including the complex interaction between the immune system, neural networks, and structural components of the respiratory system [25].

Since the traditional experimental models of allergen sensitization by injection are not similar to human disease, some newer models have been developed mimicking how real-world asthma occurs. To investigate the response to continuous antigen exposure, similar to human disease, mice were exposed to extracts of house dust mites (HDM) or ovalbumin (OVA) intranasally for five consecutive days, followed by 2 days of rest, for seven consecutive weeks [26]. Continuous exposure to HDM, unlike OVA, caused serious and persistent eosinophilic inflammation. Histological analysis of the lungs showed evidence of airway remodeling in mice exposed to HDM, with goblet cell hyperplasia, collagen deposition, and peribronchial contractile tissue accumulation. After 9 weeks without HDM exposure, inflammation of the airways completely resolved and airway hyperresponsiveness partially improved, but remodeling had not changed [26].

Currently, there is a wide range of murine models of asthma, with different underlying pathological mechanisms ranging from nonatopic disease to transgenic models. Models with overexpression of the Th2-specific transcription factor GATA3 or the Th17-transcription factor ROR*γ*t provide methods for studying* in vivo *the role of Th2 and Th17 immune response in allergic airway disease, respectively [27, 28].

These new mouse models have also become appreciated as being more complex, with mixed airway granulocytic infiltrate (eosinophilic and neutrophilic), covering the immune responses of Th2 and Th17 profile. It has been demonstrated that the pathogenicity of asthma can include the Th17 response [29]. The neutrophilic models allowed researchers to elucidate different underlying mechanisms in a disease with lower sensitivity to corticosteroids [27, 30, 31]. Thus, availability of animal models with different phenotypes and endotypes of asthma will provide the development of better treatments for humans with nonclassical atopic disease [25].

## 5. Microbiome and Asthma

The immune system is linked to the human ecosystem, which includes 10^13^–10^14^ bacteria [32]. Joshua Lederberg coined the term “microbiome” and described the microflora as part of the human metabolism. The human microbiome includes all bacteria, not only in the intestine, but also in the upper and lower respiratory tract, skin, and urinary tract. Since 2007, the Human Microbiome Project (HMP) from the National Institutes of Health (NIH) and the European Program Metagenomics of the Human Intestinal Tract (MetaHit Consortium) has sequenced the genomes of all organisms inhabiting the human body.

The relationship between exposure to microbes and asthma continues to stimulate scientific interest. Both human epidemiological and animal experimental studies have been done to better understand the interaction between the microbiome and the chronic allergic respiratory disease. Regarding human asthma, both the role of exposure to microbes in early life and the impact of bacteria colonization or infection in patients already diagnosed with asthma have been investigated [33–44].

It is important to distinguish the bacteria accurately to understand the relationship between microbiome and immune response. The diversity of known bacteria is enormous and each biological effect in respiratory disease is probably species specific.

Well-developed techniques focused on studies of bacteria are based on analysis of the 16S ribosomal RNA (rRNA) gene. This gene has properties that facilitate bacterial community analysis, such as the presence of broadly conserved sequences that flank a number of hypervariable regions. There are several higher resolution molecular tools to study the 16S rRNA diversity, including microarray phylogenetic analysis and next generation sequencing-based approaches [45–50].

## 6. Environmental Microbiome and Asthma

Environmental factors have been associated with either risk or protection for allergic sensitization and asthma. Exposure to farm animals [51–56] and raw milk consumption have been associated with decreased atopic disease development in childhood [57]. Moreover, antibiotic use during pregnancy or the first year of life has been associated with increased risk for the development of asthma [12], and Gram-negative bacteria endotoxin levels in mattress dust are inversely related to sensitization and atopic asthma in children [58]. Schaub et al., studying 84 pregnant women, noted that exposure to microbes in the prenatal period was associated with increase of Treg cells with higher expression of FoxP3 and lower secretion of Th2 cytokines in umbilical cord blood [59]. Thus normal microbiota environmental stimuli shape the expression of host genes and/or immune responses, even without modifications in the composition of the host microbiota.

## 7. Gut Microbiome and Asthma

The microbiota is an antigenic stimulus that assists in the development of the immune system in early life [54, 55]. The composition of the intestinal microbiota plays an important role in defining the phenotype of the immune response [56–59]. Different species within a particular bacterial family can also have different immune-stimulating effects, as reported in the case of bifidobacteria and lactobacilli [47, 60]. It has been shown that, among breast-fed infants,* Bifidobacterium bifidum* was the main* Bifidobacterium* species found in fecal specimens from nonallergic infants, while* B. adolescentis* and* B. longum* were more prevalent in those who developed allergy [47]. These data show that each bacterial species has a different interaction with the immune system.

Bacterial species or bacterial diversity in the gut may be implicated in the development of asthma. In a prospective study of 117 children classified by the Asthma Predictive Index (API), fecal samples taken at three weeks of age showed a higher prevalence of* Bacteroides fragilis* and other anaerobic bacteria in the API-positive in comparison with the API-negative group [48]. In a birth cohort study of 411 children at high risk for asthma, stool samples collected at one and twelve months after birth were analyzed by 16S rRNA-based denaturing gradient gel electrophoresis (DGGE). Reduced bacterial diversity was inversely associated with allergic sensitization in the first six years of life, though not with the development of asthma [38]. Evidence from studies of the environment and intestinal flora indicates that the reduction of exposure to a variety of microbes, including specific microbial consortia, has negative implications for the health of the immune system that includes risk for allergy and asthma [61].

## 8. Respiratory Microbiome and Asthma

The idea that bronchial infection may underlie asthma was fostered by epidemiological studies that have reported an association between community outbreaks of* Chlamydophila pneumoniae* and the occurrence of late-onset asthma [62]. Analyses of sequencing techniques based on microarray and 16S rRNA are able to define the profile of the flora in asthmatic airways. Studies have shown that the composition of the bacterial microflora detected from the lower respiratory tract differs in persons with asthma compared to healthy individuals [43, 44, 63].

The predominant bacterial communities associated with asthma were mostly represented by groups of bacteria that are not members of the dominant oral microbiome [43, 63]. The members of the phylum Proteobacteria, in particular* Haemophilus *spp., have been more commonly identified from bronchial brushing or lavage from individuals with airway disease. Members of the phylum Bacteroidetes, such as* Prevotella *spp., were more often found in samples from healthy individuals. Specific bacterial families observed more frequently among patients with asthma include Enterobacteriaceaeand Neisseriaceae [63]. However, some bacteria may have protective effects. For example, nasal administration of* Lactobacillus rhamnosus* induced protection against respiratory syncytial virus infection in mice [64].

Recently, a study analyzing the fungal composition of induced sputum samples showed several differences in species composition between asthma patients and controls [65].* Malassezia pachydermatis, *a fungus that was previously associated with atopic dermatitis, was one of the fungi most frequently found in samples of asthmatic patients. However, this finding is not detrimental and there is a need for more studies regarding fungal sequence databases. So far, the role of fungal colonization in asthma is still unclear [66].

As clinical trials assessing microbiome and human asthma are still not available, data from animal models are the basis for better understanding the role of bacteria in the respiratory disease. Data available regarding the interaction between the microbiome and human asthma are summarized in Table 1.

## 9. Animal Models and the Microbiome

Recent studies in animal models demonstrate that gut microbiota is an important driver of immune response that can modulate allergic inflammation in the airways and features of asthma [67–69]. The germ-free mice compared to specific pathogen-free mice exhibit increased accumulation of mucosal invariant natural killer T cells (iNKT) in the colonic lamina propria as well as in the lung. These germ-free mice, in an ovalbumin model of allergic asthma, presented increased airway resistance and eosinophils count in bronchoalveolar lavage and tissue. Feeding neonate germ-free mice with conventional microbiota, but not adults, minimized the accumulation of iNKT cells in the lungs and attenuated allergic inflammation and asthma [67]. In another study, vancomycin administration to neonatal mice led to reduced bacterial diversity and similar increased susceptibility to allergic asthma in a murine model [69].

Several studies have demonstrated that oral administration of bacterial species with immunomodulating properties can modulate features of allergic asthma in the lungs. Oral supplementation with* Bifidobacterium* species and specific* Lactobacillus* led to reduced production of Th2 cytokines and eosinophilic inflammation and promoted Treg and Th17 populations [68–73].

The probiotic* Lactococcus lactis* has been used in a model of asthma induced by HDM and induced inhibition of Th2 population, preventing the development of allergic inflammation [74]. Findings of these and other studies suggest that interventions in intestinal microbiome during a specific period of time may alter susceptibility or attenuate allergic asthma.

Heat-shock protein X (HspX) purified from* Mycobacterium tuberculosis* shifted the immune response to the Th1 profile in a mouse model of OVA-induced asthma. Furthermore, HspX enhanced OVA-induced decrease of regulatory T cells in the mediastinal lymph nodes. These findings could provide new insights into the immunotherapeutic role of HspX with respect to its effects on a murine model of asthma [75].

Administration of* Bacillus Calmette-Guérin* (BCG) in models of asthma led to significant decrease in the levels of OVA-specific IgE, eosinophils in lung tissue, and eosinophils, neutrophils, and lymphocytes in bronchoalveolar lavage [76]. Moreover, BCG resulted in a significant increase in lung compliance and conductance in the OVA-plus-BCG group [76]. Combined BCG vaccine with interleukin-12 had an even greater impact than the BCG alone in the experimental allergic inflammation of the airways [77].

Colonization with* Streptococcus pneumoniae* (*S*.* pneumoniae*) is associated with an increased risk for recurrent wheeze and asthma. But killed* S*.* pneumoniae* showed some potential as an effective immunomodulatory therapy in a murine model of asthma [78]. We summarized the results of studies assessing interaction between bacterial agents or antibiotic exposure and murine models of asthma in Table 2.

More than 30 studies were published on animal models exploring helminthic infection as a protective factor against allergy. The majority of these experiments, which were conducted with a wide range of hypersensitivity models (17 of them were on asthma models), demonstrated that helminths protect against development of allergic disease. In 26 studies, the effect of helminthic infection after sensitization was analyzed. In four other studies, animals were previously infected with helminths and then sensitized. Two out of these 4 studies showed evidences of protection [79]. Other studies were performed with helminth antigens;* pseudocoelomic* fluids, excretory-secretory products, and whole-worm antigen preparations were capable of protecting the host against the development of allergy. Unfortunately 2 prospective double-blind trials of helminth infection and allergic disease in human subjects did not show benefits [79].

Finally, a study recently published [80] showed that exposure to house dust mite in early life influences the lung microbiome establishment in a BALB/c model of respiratory allergic disease. Changes in lung bacterial load and community composition after birth were associated with decreased airway responses to aeroallergen exposure [80]. Interestingly, this tolerance was not associated with the existing presence of high numbers of regulatory T cells in the lung at birth, but rather with the development of a different T regulatory cell subset that seemed to require microbial presence during a critical early window after birth. These findings suggest that establishment of a lung microbiome occurs and is a dynamic process after normal birth and that the relationship between microbiota and lung-specific immune development is not static.

## 10. Conclusion

The number of publications regarding microbiome association with asthma and allergy has been rising significantly in the last years, but differences in sampling methods, experimental protocols, and bioinformatic data analysis hamper comparisons.

The patient airway microbiome includes species with pathogenic potential, as well as species with potential immunomodulatory or metabolic properties relevant to asthma specific mechanisms. The interdependency of bacterial, viral, and fungal challenges and the bidirectional crosstalk with the human immune system seems too extensive. A better understanding of the human microbiome and its impact on the host health status might lead to more effective strategies in disease prevention and treatment.

## Figures and Tables

**Table 1:** Microbial exposures and asthma.

Exposure	Microbial exposure assessment	Effect	Endpoint	References
Environment	Exposure to animals (dogs, cats, and farm animals)	Decrease	Allergy or asthma	[51–56]
	Raw milk consumption	Decrease	Atopy or asthma in childhood	[57]
	Antibiotic use during pregnancy or first year of life	Increase	Risk of asthma	[42]
	Gram-negative bacteria's endotoxin levels in mattress dust	Inverse relation	Atopic asthma and sensitization in children	[58]
	Prenatal microbial exposure	Increase	Cord blood Treg cell counts FOXP3 expression	[59]
		Decrease	TH2 cytokine secretion	

Digestive tract	*Bifidobacterium bifidum *	More prevalent	Stool samples of nonallergic infants	[47]
	*Bifidobacterium adolescentis *and *B*. *longum *	More prevalent	Stool samples of allergic infants	
	*Bacteroides fragilis *	High prevalence	Stool samples of children with positive prediction for asthma	[48]
	Bacterial diversity	Inverse association	Allergic sensitization	[38]

Respiratory tract	Bronchial infection by *Chlamydophila pneumonia *	Association	Asthma development	[40]
	Microbiota species in lower respiratory tract	Different	Asthmatic versus healthy subjects	[43, 44, 63]
	*Haemophilus *spp. in airways	More frequent	Subjects with airway diseases	[63]
	*Prevotella *spp.in airways	More frequent	Healthy subjects	
	Enterobacteriaceae and Neisseriaceae	More frequent	Asthmatic patients	[63]
	*Malassezia pachydermatis *	Frequent	Asthmatic patients	[65]

**Table 2:** Models of asthma and microbiome.

Mice strain/antigen	Microbial or exposure evaluation	Effect	Endpoint	References
C57BL/6/nonantigen	Germ-free mice	Increase	Airway resistance and eosinophils in airways	[67]

C57BL/6/OVA	Vancomycin to neonatal mice	Decrease	Bacterial diversity	[69]
		Increase	Inflammation in allergic asthma	

BALB/c/OVA	Oral supplementation with *Lactobacillus reuteri *	Decrease	Allergic airway response	[70]

BALB/c/OVA	Oral supplementation with *Bifidobacterium longum *	Decrease	Inflammation in allergic asthma	[71]

BALB/c/OVA	Oral supplementation with *Enterococcus faecalis* FK-23	Suppression	Th17 immune response	[73]

BALB/c/HDM	*Lactococcus lactis *	Inhibition	Th2 response	[74]

BALB/c/OVA	Heat-shock protein X (HspX) purified from *Mycobacterium tuberculosis *	Regulation	Th1 immune response	[75]

BALB/c/OVA	BCG	Decrease	Specific-IgE levels, eosinophil, neutrophil, and lymphocyte counts in BAL	[76]

BALB/c/OVA	BCG and interleukin-12 vaccination	Decrease	Allergic airway inflammation	[77]

BALB/c/OVA	Killed *Streptococcus pneumoniae *	Protection	Asthma	[78]
